# Invagination intestinale aigue sur sonde de jéjunostomie: cause rare d'occlusion intestinale

**DOI:** 10.11604/pamj.2014.17.137.3858

**Published:** 2014-02-27

**Authors:** Khalid El Haoudi, Said Ait Laalim, Karim Ibn Majdoub Hassani, Imane Toughrai, Khalid Mazaz

**Affiliations:** 1 Service de chirurgie viscérale, CHU Hassan II, Fès, Maroc

**Keywords:** Invagination intestinale aiguë, sonde de jéjunostomie, désinvagination, Acute intussusception, jejunostomy tube, intussusception

## Abstract

L'invagination intestinale aiguë (IIA) secondaire à une cause inhabituelle est une complication rare et une urgence abdominale. Elle pose un problème diagnostique et thérapeutique. Nous rapportons le cas d'un patient âgé de 45 ans, qui présentait une sténose bulbaire peptique résistante au traitement médical. Le patient a été opéré avec présence en per opératoire d'un estomac de stase, en amont d'une sténose bulbaire infranchissable. Une jéjunostomie d'alimentation à la Witzel a été réalisée vu l’état général très altéré du patient. L’évolution était marquée par l'installation d'une invagination sur sonde de jéjunostomie ayant nécessité une intervention chirurgicale avec des suites simples. L'amélioration de la prise en charge et du pronostic de l'IIA secondaire à une cause inhabituelle nécessitant un diagnostic précoce. Le traitement de cette forme particulière d'invagination est presque exclusivement basé sur la chirurgie.

## Introduction

L'invagination intestinale aiguë du grêle se définit par le télescopage d'un segment proximal dans la lumière du segment adjacent. Elle constitue une complication très rare chez l'adulte et survient sur des lésions infectieuses ou organiques [1]. L'invagination intestinale aiguë du grêle se manifeste par une occlusion haute du grêle. Le diagnostic peut être posé soit en préopératoire, par une échographie abdominale ou une tomodensitométrie, soit en per opératoire. Le traitement est le plus souvent chirurgical.

## Patient et observation

Patient âgé de 45 ans, qui présente une sténose bulbaire peptique résistante au traitement médical. Le patient a été opéré avec présence en per opératoire d'un énorme estomac de stase, complètement atone et une sténose bulbaire infranchissable. Une jéjunostomie d'alimentation à la Witzel a été réalisée vu l’état de dénutrition importante du patient ne permettant pas une résection gastrique ou une gastro-entéro anastomose.

Les suites post opératoires ont été marquées par la survenue à j+5 d'une occlusion grêlique. L'examen montrait un patient conscient, fébril à 38C, eupneique, stable sur le plan hémodynamique avec des plis de déshydratation et un abdomen très distendu et légèrement sensible en totalité. Le bilan biologique montrait une hyperleucocytose à 16 500 /uL, CRP à 38 mg/l, Hb=10,5g/dl, une hypoprotidémie à 24 g /l et une hyponatrémie à 125 mEq/L. L'abdomen sans prépa- ration montrait des niveaux hydro-aériques de type grêlique.

**Figure 1:**
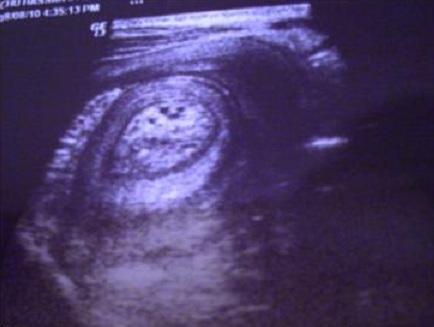
Aspect échographique en sandwich de l'invagination intestinale

**Figure 2:**
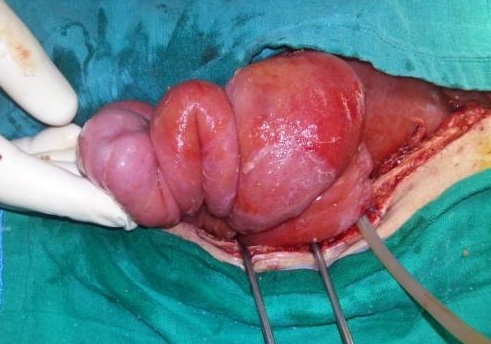
Aspect per opératoire de l'invagination intestinale sur la sonde de jéjunostomie

**Figure 3:**
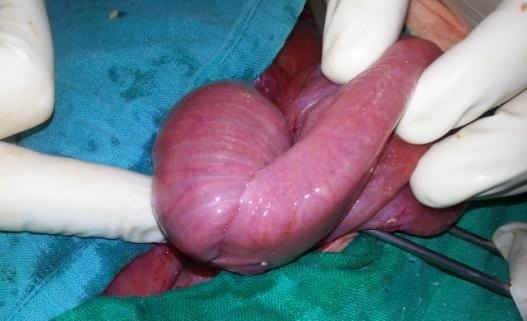
Aspect per opératoire de l'invagination intestinale après retrait de la sonde de jéjunostomie

L’échographie abdominale objectivait la présence au niveau du flanc gauche d'une image en cocarde hypo échogène, hétérogène mesurant 48 mm de diamètre évoquant une invagination intestinale aigue. Sur l'axe longitudinal on notait une image en « sandwich » à contenu intestino-épiploïque (Figure 1). L'intervention chirurgicale était indiquée et le patient a été repris chirurgicalement à J+8, d'où la découverte d'une invagination grêlo- grêlique proximale située à 50 cm de l'angle de Treitz étendue sur 15 cm sur sonde de jéjunostomie avec un grêle viable (Figure 2, Figure 3).

Le geste a consisté en une désinvagination manuelle et une reconfection d'une deuxième jéjunostomie, avec des suites post opératoires qui étaient simples et la reprise du transit était à j+2.

## Discussion

Les invaginations intestinales sont classées en fonction de leur localisation dans le tractus gastro-intestinal: entériques, colo-coliques, iléo coliques, colorectales et recto-rectales [1]. Elles peuvent être antérogrades ou rétrogrades. L’âge moyen de survenue est de 50 ans et le sexe ratio est de 1 [1, 2]. Le tableau clinique est peu spécifique et les symptômes les plus fréquents sont des douleurs abdominales suivies d'une occlusion grélique comme chez notre patient. Il existe Certains facteurs de risque représentés par le spasme et le péristaltisme anormal du tube digestif, la formation d'adhérences postopératoires, la stimulation causée par la manipulation chirurgicale et l'inflammation postopératoire [3].

La présence de selles sanglantes, de douleurs abdominales, et d'une masse abdominale palpable est très évocatrice [4]. La survenue d'une invagination intestinale peut être expliquée sur le plan physiopathologique par la perturbation du péristaltisme en rapport avec la présence d'un matériel dans la lumière intestinale ou d'une atteinte de la paroi intestinale [5]. Par conséquent, l'invagination est souvent mal diagnostiquée chez l'adulte [6].

Dans notre cas le diagnostic de l'invagination était difficile et a été posé en per opératoire. Dans la littérature le diagnostic est suspecté en préopératoire chez seulement 14% à 75% des patients [7]. Sur le plan radiologique la tomodensitométrie abdominale est l'examen le plus sensible pour confirmer le diagnostic d'invagination intestinale. L’échographie peut poser le diagnostic d'invagination intestinale chez l'adulte en montrant des images sous forme de sandwich sur l'axe longitudinal. Les limites de cet examen sont l'obésité et la distension importante [8]. Sur le plan thérapeutique il y a une controverse sur la réduction de l'invagination avant la résection. L'invagination du grêle doit être réduite uniquement chez les patients chez qui un diagnostic bénin a été fait en préopératoire ou chez les patients dont la résection peut provoquer un syndrome de grêle court en raison de la forte incidence de cancer associé et qui varie de 1% à 40% [9].

Chez notre patient on a choisi la désinvagination sans résection vu le constat per opératoire évident [9]. Il est recommandé de respecter certaines règles nutritionnelles décrites par l'American Society of nutrition pour le bon fonctionnement d'une nutrition entérale par jéjunostomie. Il existe une stratégie de prévention qui regroupe les éléments suivants: démarrer une alimentation entérale après le reprise du transit, multiplier les trous latéraux faits dans la sonde de jéjunostomie pour diminuer la pression, commencer par un débit à un rythme de 10-20 ml/h et suturer largement le jéjunum.

## Conclusion

Les invaginations intestinales sur sonde de jéjunostomie est une urgence abdominale rare. L'inflammation postopératoire, le spasme anormal du tube digestif et la sonde elles même représentent les principaux facteurs de risque. La réduction de cette forme particulière d'invagination est essentiellement chirurgicale.

## References

[1] Pua U (2011). Multisegment jejunojejunal intussusception in gastrojejunostomy. Med J Aust..

[2] Hasnai H, Mouaqit O (2013). Une observation rare d'occlusion intestinale: double invagination intestinale sur sonde de jéjunostomie. J Afr Hépatol Gastroentérol.

[3] Pelosof L, Ringold DA, Kuo E (2007). Retrograde jejunogastric intussusception caused by a migrated gastrostomy tube. Endoscopy..

[4] Ishii M, Yakabe M, Teramoto S (2007). A 94-year-old woman with nontuberculous mycobacterium who developed small intestinal intussusception associated with a percutaneous endoscopic jejunostomy tube. Nihon Ronen Igakkai Zasshi..

[5] Ragunath K, Roberts A, Senapati S (2004). Retrograde jejunoduodenal intussusception causedby a migrated percutaneousendoscopic gastrostomy tube. Dig Dis Sci..

[6] Athanasios M, Anneza Y, Lazaros S (2009). Intussusception of the bowel in adults: A review World. J Gastroenterol..

[7] Goutos I, O'Sullivan AW, Myint F (2008). Idiopathic jejunalintussusception in an adult. J Med Sci.

[8] Ana B, Hamilton B, Carlos P (2009). Jejuno-jejunal invagination caused by epithelioid sarcoma: a case report. J Med Case Reports..

[9] Guo-Shiou Liao, Huan-Fa Hsieh, Meng-Hang Wu (2007). Knot formation in the feeding jejunostomy tube: A case report and review of the literature. World J Gastroenterol.

